# Sensitive, Noninvasive Detection of Lymph Node Metastases

**DOI:** 10.1371/journal.pmed.0010066

**Published:** 2004-12-28

**Authors:** Mukesh G Harisinghani, Ralph Weissleder

**Affiliations:** 1Massachusetts General Hospital and Harvard Medical School, BostonMassachusettsUnited States of America; Technical University MunichGermany

## Abstract

**Background:**

Many primary malignancies spread via lymphatic dissemination, and accurate staging therefore still relies on surgical exploration. The purpose of this study was to explore the possibility of semiautomated noninvasive nodal cancer staging using a nanoparticle-enhanced lymphotropic magnetic resonance imaging (LMRI) technique.

**Methods and Findings:**

We measured magnetic tissue parameters of cancer metastases and normal unmatched lymph nodes by noninvasive LMRI using a learning dataset consisting of 97 histologically proven nodes. We then prospectively tested the accuracy of these parameters against 216 histologically validated lymph nodes from 34 patients with primary cancers, in semiautomated fashion. We found unique magnetic tissue parameters that accurately distinguished metastatic from normal nodes with an overall sensitivity of 98% and specificity of 92%. The parameters could be applied to datasets in a semiautomated fashion and be used for three-dimensional reconstruction of complete nodal anatomy for different primary cancers.

**Conclusion:**

These results suggest for the first time the feasibility of semiautomated nodal cancer staging by noninvasive imaging.

## Abstract 2

Injection of targeted nanoparticles, combined with magnetic resonance imaging, allows identification and detailed three dimensional placement of malignant lymph nodes

## Introduction

Most primary malignancies spread systemically via lymphatic dissemination [1]. For example, the finding of axillary nodal metastases predicts a much shorter disease-free survival in breast cancer [2]. The total nodal tumor burden (number of affected nodes and metastatic tumor volume) affects prognosis even more severely [3]. Accurate lymph node staging also remains a cornerstone in choosing the most appropriate therapy for a given stage. Therapeutic intervention of metastatic lymph nodes [4], prophylactic radiation of frequently affected drainage routes [5], and systemic therapies [6] all have been shown to improve survival. Genetic profiles identifying metastatic tumors [7], serum biomarkers, and proteomic profiles are currently being developed to identify patients at risk [8,9]. No direct genetic profile, however, has been demonstrated to date to accurately predict the presence of human nodal metastases in a given patient. Rather, surgical approaches, such as sentinel lymph node biopsy or lymph node dissection, are still commonly used. Careful histological analysis includes mapping, bisectioning, and rapid staining in the frozen tissue laboratory. Higher diagnostic accuracies can be achieved by serial sectioning (50 μm) and by immunohistochemical staining [10,11].

Noninvasive imaging studies are commonly used during the workup of primary malignancies. Typically, lymph nodes are diagnosed by tomographic techniques (computed tomography [CT], magnetic resonance imaging [MRI]) as malignant when their short axis is >10 mm in size [12]. Such size criteria, however, have been shown to be unreliable [13]. Similarly, the detection of cancer in nonenlarged (occult) nodes is often quite low by positron-emission tomography (PET) and single photon emission computed tomography imaging. For example, small nodal metastases (< 5 mm) are often missed by PET imaging in patients with breast cancer [14]. More recently, it has become possible to image anatomic regions at submillimeter resolutions by MRI, with excellent spatial coverage and reduced motion artifacts. The development [15,16] and clinical introduction of lymphotropic magnetic nanoparticles has been shown to significantly improve diagnostic accuracies of MRI for nodal staging (LMRI) in prostate cancer [17]. These nanoparticles serve as probes for lymphatic anatomy and function and enhance tumor detection through abnormal distribution patterns in malignant nodes [17,18].

Despite the advances of LMRI for cancer staging, image analysis has been challenging and occasionally controversial. Traditional analysis has been based on a reader's identification of certain structural abnormalities that can be variable, given differences in acquisition parameters and interpretation criteria [19,20,21]. Furthermore, it has been challenging to quickly and accurately analyze large datasets generated by LMRI.

The goal of the current study was to develop and test technologies that would vastly improve the accuracy of current LMRI nodal staging. Specifically we set out to (a) determine whether unique magnetic parameters existed and could be used for semiautomated image analysis and (b) whether the technique could be applied to different primary cancers. Here we provide the first comprehensive analysis of tissue parameters validated against histopathology as an end point.

## Methods

### Study Design

The Institutional Review Board approved the current study and all patients signed informed consent. The study was divided into a learning (*n* = 97 lymph nodes with known histopathology) and a test dataset (*n* = 216 lymph nodes with known histopathology; Table 1). Assignment into datasets was done in temporal fashion. The learning dataset represented retrospective cases at outset of the study, and the test dataset represented prospective cases collected during a 1-y interval. In the learning set, 55% of the nodes were benign, and 45% of the nodes were malignant. The learning dataset was obtained from 36 patients (24 male, 12 female, age 28–85 y, mean 59.7 y) with histologically proven primary genitourinary malignancies (prostate, 21; bladder, 9; testes, 5; ureter, 1). All patients completed the MRI study and then underwent surgical resection (*n* = 26) and/or nodal biopsy (*n* = 10). The investigated nodes had a mean short axis diameter of 10.5 mm (range 3–39 mm).

**Table 1:**
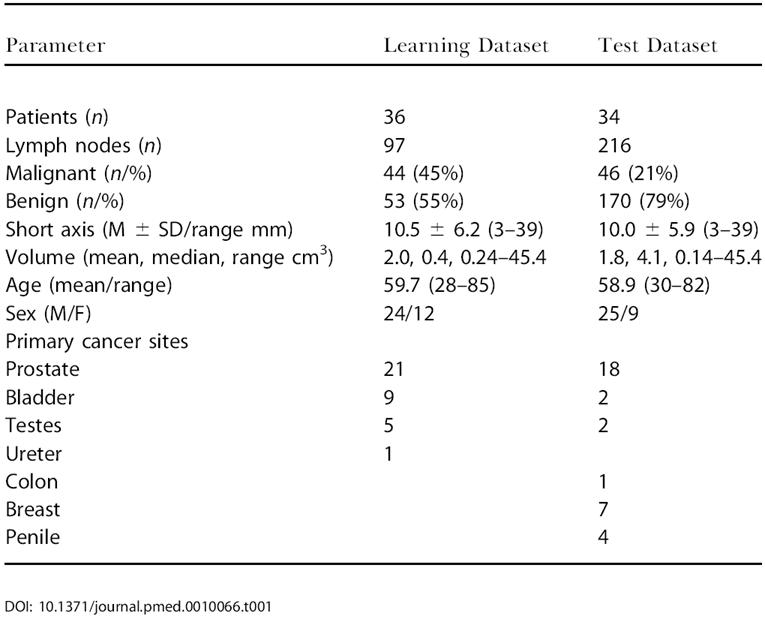
Overview of Patient Datasets

The test dataset was obtained from 34 patients (25 male, nine female, age 30–82 y, mean 58.9 y) with histologically proven malignancies from different primaries (Table 1), including prostate (*n* = 18), breast (*n* = 7), penile (*n* = 4), bladder (*n* = 2), testes (*n* = 2), and colon (*n* = 1). Seventy-nine percent of the nodes were benign and 21% of the nodes were malignant. The nodes in the test dataset had a mean short axis diameter of 10.0 mm (range 3–39 mm). Both datasets included the full spectrum of normal nodes to completely replaced nodes.

### MRI

MRI was performed at 1.5 T (System 9X, General Electric Medical Systems, Milwaukee, Wisconsin, United States) using phased-array coils. All images were archived on DICOM PACS servers (MIPortal, CMIR and Siemens Medical Systems, Erlangen, Germany; and Impax RS 3000, AGFA Technical Imaging Systems, Richfield Park, New Jersey, United States) for subsequent analysis. Images of the pelvis (*n* = 56) extended from the pubic symphysis to just above the level of aortic bifurcation. In patients with primary testicular cancers (*n* = 7) imaging was extended superiorly to include the renal hilum and retroperitoneum. In patients with breast cancer (*n* = 7) we obtained MR images of the bilateral axillae, including the internal mammary and supraclavicular regions. All patients were imaged with identical pulse sequences and timing parameters. Imaging was performed before and 24 h after intravenous ferumoxtran-10 administration (Combidex, Advanced Magnetics, Cambridge, Massachusetts, United States; 2.6 mg Fe/kg diluted in normal saline and infused over a 20-min period using a 5-μm filter).

The acquired pulse sequences included (a) axial T2-weighted fast spin-echo (TR/TE, 4500/80; flip angle, 90°; field of view, 24–28 cm; slice thickness, 3 mm; matrix, 256 × 256; number of excitations, 2; in-plane resolution, 1.2 mm); (b) a T1-weighted two-dimensional gradient-echo sequence obtained in different anatomical planes (TR/TE 175/1.8; flip angle, 80°; field of view, 22–30 cm; slice thickness, 4 mm; matrix, 128 × 256; in-plane resolution, 2.0 mm); (c) an axial T2-weighted dual TE gradient-echo (TR/TE 2100/14–24; flip angle, 70°; field of view, 26–28 cm; slice thickness, 3 mm; matrix, 160 × 256; in-plane resolution, 1.7 mm); and (d) a three-dimensional (3D) T1-weighted gradient echo sequence; TR/TE 4.5–5.5/1.4; flip angle, 15°; field of view, 24–28 cm; slice thickness, 1.4 mm; matrix, 256 × 256; in-plane resolution, 1.0 mm).

The above listed imaging sequences and parameters had previously been optimized to reduce motion artifacts, maximize signal-to-noise ratio (SNR), and provide diagnostically useful images of the pelvis, abdomen, and chest within clinically acceptable time limits. The T2-weighted fast spin-echo sequence, in (a) above, was primarily used for qualitative nodal detection, and hence a square pixel with more than one acquisition was obtained. The two-dimensional axial T1-weighted gradient-echo sequence, in (b) above, was chosen to achieve adequate anatomical coverage within a short imaging time. The axial dual-echo gradient-echo sequence, in (c) above, was developed specifically for this project to provide artifact-free datasets for quantitative image analysis. A matrix size of 160 × 256 was chosen for this sequence to achieve a balance between the upper limits for imaging time while reducing image noise. Finally, a 3D T1-weighted sequence was obtained, in (d) above to provide a dataset for vascular maximum intensity projection (MIP) reconstructions.

### Quantitative Image Analysis

All image analysis was performed on archived DICOM images using different software packages (e.g., custom-built software such as CMIR-Image, MGH, Boston, Massachusetts, United States; Syngo, Siemens Medical Systems; Advantage Windows, General Electric Medical Systems). Lymph nodes were identified by readers who manually placed kernels onto each node for automated boundary detection and calculation of nodal dimensions and volumes. The thus identified regions of interest (ROIs) encompassed the entire lymph node (not only portions of it) and were used for quantitative signal-intensity (SI) measurements (see Table 2). Serial measurements of nodal dimensions on different pulse sequences or time points varied less than 2%.

**Table 2:**
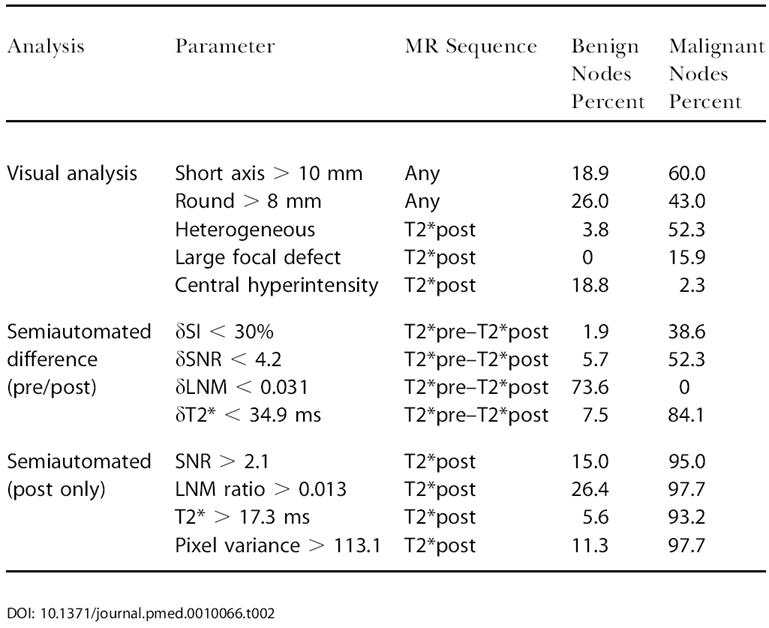
Frequency of Imaging Parameters in Learning Dataset

A number of quantitative tissue parameters were calculated either as differences between pre- and postcontrast scans (δ) or as single-value analysis on postcontrast scans (see Table 2). The lymph node/muscle (LNM) ratio was calculated by dividing signal intensities of an entire lymph node by that of adjacent muscle using a similar-sized ROI, drawn manually. The nodal SI change was calculated by obtaining SI before and after contrast administration. The nodal SNR was calculated by obtaining SD/SD_noise_. The T2* was calculated in nodal ROIs on dual TE images using CMIR-Image. T2* maps were constructed by performing fits of a standard exponential relaxation model (S = Ke^–TE/T2*^) to the data on a pixel-by-pixel basis. Only pixels with intensity greater than a threshold level (2X of noise) were considered during the fitting process. Pixel variance was obtained from post-MR images. Comparative visual analysis included short axis measurements, and identification of heterogeneity, large focal defects, and central hyperintensity, according to criteria previously established [12,17].

To determine the diagnostic accuracy of the different tissue parameters in the learning dataset, we determined sensitivity, specificity, and predictive values for each parameter alone and in combination (Table 3). The most discriminatory parameters were then applied to the test dataset (Table 4).

**Table 3:**
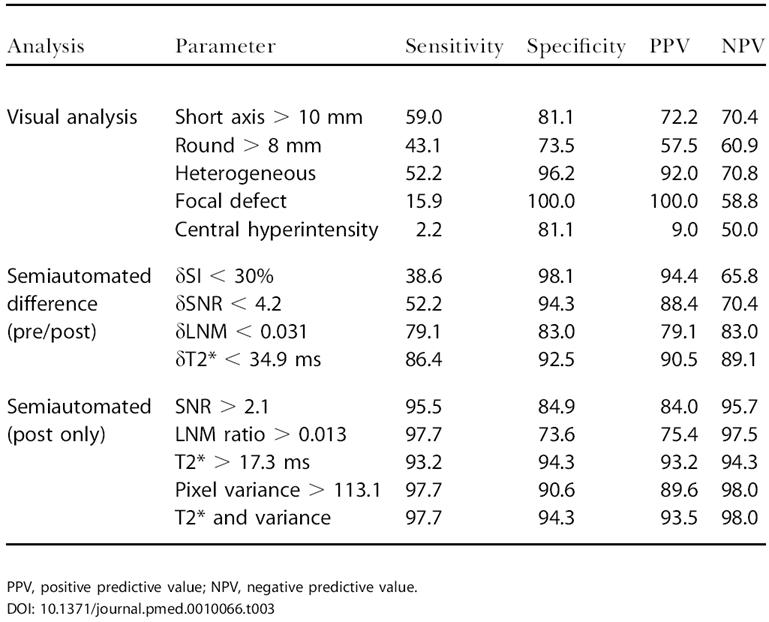
Discriminatory Power of Imaging Parameters in Learning Dataset

PPV, positive predictive value; NPV, negative predictive value

**Table 4:**
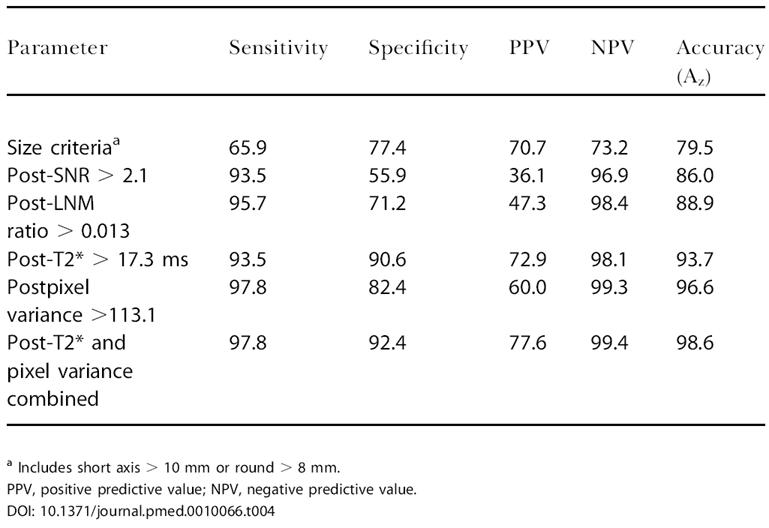
Application of Quantitative Parameters to Test Dataset (*n* = 216)

^a^ Includes short axis > 10 mm or round > 8 mm

PPV, positive predictive value; NPV, negative predictive value

In the final set of semiautomated image analysis, 3D reconstructions were obtained for nodal mapping onto vascular anatomy using MIP projections. While the MIP projections do not aid in the differentiation between malignant and benign lymph nodes, they are invaluable in providing anatomic content to the dozens of lymph nodes identified. In particular, MIP images were generated interactively from postcontrast, fat-saturated, volumetric interpolated breath-hold images to outline vascular anatomy. The evaluated lymph nodes characterized as benign or malignant (by T2*/variance analysis) were then superimposed on the volumetric 3D images, using customized software (Advantage Windows, General Electric Medical Systems).

### Statistical Analysis

**Figure 1:**
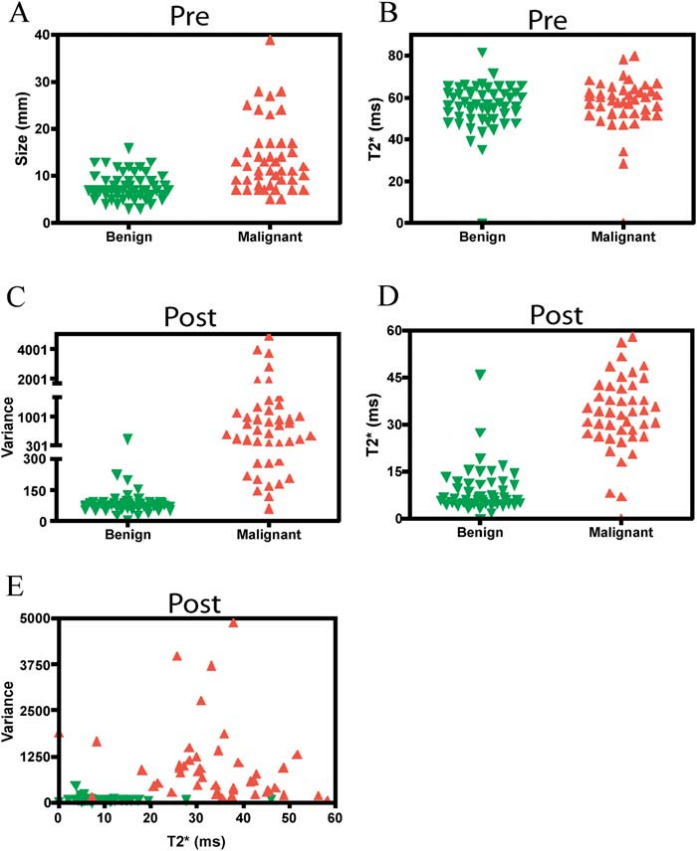
Tissue Parameters in Learning Dataset Nodal tissue parameters for benign and malignant nodes are shown before (A and B) and after (C–E) intravenous administration of magnetic nanoparticles. Note the insensitivity of conventional MRI (A and B), better separation using single-value analysis (C and D) and excellent separation using two-value analysis (E).

Data were expressed as mean ± standard deviations (SD) and medians. All statistical testing was performed using GraphPad Prism (GraphPad Software, San Diego, California, United States). The significance between two individual groups was determined using the nonpaired Student's *t*-test (e.g., benign and malignant datasets in Figure 1). For the more discriminatory datasets alternative-free-response receiver operating characteristic curves were plotted. Ratios for cut-off single-value parameters were defined to yield highest sensitivity and specificity. Accuracy for a given parameter was expressed as the area under the curve (A_z_), and values are summarized in Table 4.

### Histology

All lymph nodes were sampled histologically within 2 wk of the MRI (mean: 6 d; range: 2–14 d). The analysis was done in surgically resected lymph nodes (*n* = 55; both benign and malignant nodes) or in fine needle aspirates and core biopsies (*n* = 15; malignant nodes only), implementing careful mapping procedures to correlate nodes. Surgically excised nodes were sectioned at 10–20 μm intervals after bihalving and were stained with hematoxylin-eosin.

## Results

### Learning Dataset

The learning dataset consisted of 97 histologically validated lymph nodes from 36 patients with different primary malignancies (see Table 1). The mean short axis diameter was 10.5 mm (range 3–39 mm) with 56 of the 97 nodes (58.3%) measuring less than 10 mm, that is, below the traditional imaging cutoff for malignancy (“occult nodes”). Table 2 summarizes the incidence of different visual, comparative (before and after contrast administration), and semiautomated (postcontrast administration only) parameters in the two different groups. Figure 1 is a graphical representation of overlaps between malignant and benign groups for different parameters listed in Table 2. Table 3 summarizes sensitivities, specificities, and predictive values for the different quantitative imaging parameters. Sensitivities of metastasis detection by visual image analysis ranged from 50%–94%, however, often with lower specificities. Volumetric measurements, in particular, were insensitive markers of malignancy in nonenlarged nodes (see Table 3).

In contradistinction, image analysis of pre- and postcontrast image sequences resulted in higher specificities and sensitivities (see Table 3). Comparative differences between benign and malignant nodal groups were highest for T2* and pixel variance measurements (see Table 3). Of all the semiautomated parameters tested alone, T2* measurements showed the highest sensitivity (93%; 95% confidence interval: 82%–98%) and specificity (94%; 95% confidence interval: 84%–99%) in the learning dataset (see Figure 1 and Table 3).

Of all the semiautomated parameters tested in combination, T2* measurements combined with pixel variance analyses postcontrast showed the highest sensitivity (98%; 95% confidence interval: 88%–99%) and specificity (94%; 95% confidence interval: 82%–98%) in the learning dataset (Figure 1E). Using the dual-value analysis, there was one malignant outlier in the benign dataset (the lymph node was 3 mm in overall size, with few malignant cells seen on histology, and probably too small for analysis) and two benign outliers in the malignant dataset (both these nodes showed hyalinosis replacing more than 50% of the nodal architecture).

### Test Dataset

To determine whether feature extraction would be accurate for prospective nodal staging, we utilized the above criteria against a larger test dataset encompassing 216 validated lymph nodes from 34 patients, including different primaries (see Table 1). The sensitivity, specificity, and predictive values of the most discriminatory parameters of this prospective analysis are summarized in Table 4. We primarily focused on semiautomated image analysis of postcontrast scans because of the high sensitivity and specificity determined in the learning dataset. T2* measurements showed a sensitivity of (93%; 95% confidence interval: 82%–99%) and a specificity of (91%; 95% confidence interval: 85%–96%). Combined T2* and pixel variance analysis achieved a sensitivity of 98% (95% confidence interval: 88%–99%) and a specificity of 92% (95% confidence interval: 87%–96%) comparable to that of the learning set and much superior to currently used size criteria.

Using the dual-value analysis, there were two malignant outliers in the benign dataset (both of these nodes were less than 3 mm in overall size and probably too small for analysis—similar to the learning dataset) and three benign outliers in the malignant dataset (two of these nodes had hyalinosis replacing more than 50% of the nodal architecture and one had macrocalcifications). More important, all the misclassified nodes occurred in individual patients rather than in the same patient and, hence, did not affect the overall nodal staging on a patient-by-patient basis in this dataset.

### Image Reconstruction

**Video 1:** Automated 3D Reconstruction of Pelvic Nodal Anatomy

**Figure 2:**
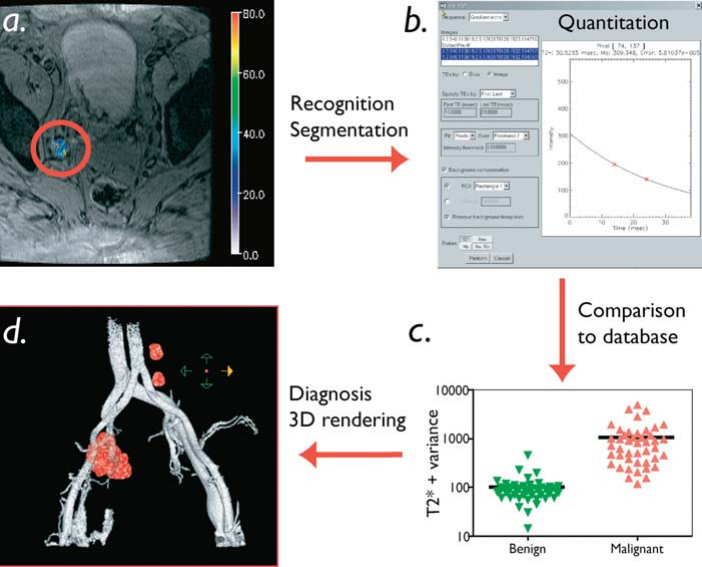
Steps in Semiautomated Image Analysis Semiautomated image analysis involves recognition and automated segmentation of each lymph node (A), quantitation of magnetic tissue parameters (T2*, variance of pixel values; [B]), comparison of extracted tissue parameter to a database (C), and 3D reconstruction of nodal anatomy onto vascular anatomy (D).

**Figure 3:**
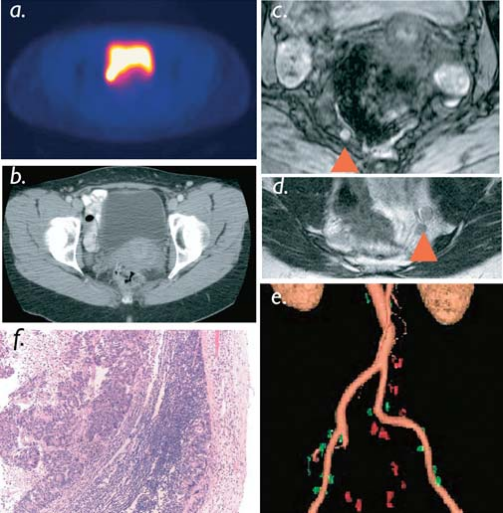
Pelvic Nodal Staging Nodal staging in patient with colorectal cancer. A PET scan using ^18^FDG as a tracer (A) and a CT scan (B) were interpreted as negative for nodal metastases. LMRI identified six small pelvic lymph nodes ([C] and [D]; red arrowheads), which had magnetic parameters of malignancy. Semiautomated reconstruction (E) identifies multisegmental metastases, subsequently proven histologically (F). For 3D reconstruction of pelvic nodal anatomy see Video 1.

**Figure 4:**
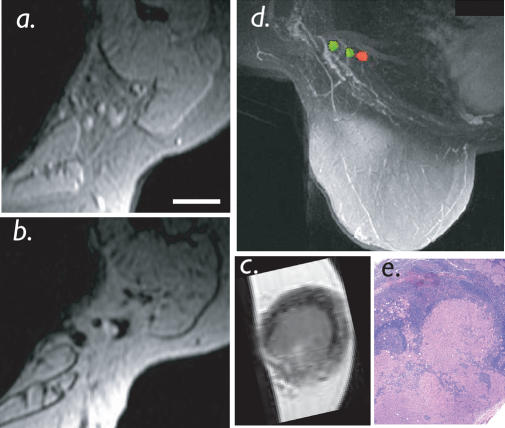
Breast Cancer Mapping Patient with breast cancer prior to sentinel lymph node biopsy. (A) Conventional axillary MRI shows nonenlarged lymph nodes that do not meet the size criteria of malignancy (white bar = 5 mm). (B) Following intravenous administration of nanoparticles, a single 3-mm intranodal metastasis was correctly identified. (C) Ex vivo MRI of sentinel node specimen confirms metastasis. (D) Semiautomated nodal analysis and reconstruction correctly juxtaposed solitary lymph node metastases adjacent to two normal lymph nodes. (E) Correlative histopathology confirms the diagnosis. For 3D reconstruction of axillary nodal anatomy see Video 2.

Utilizing semiautomated feature extraction to identify lymph nodes and image analysis (based on T2* and pixel variance), we subsequently proceeded to map individual lymph nodes onto vascular anatomy in the different anatomic drainage patterns. Figure 2 summarizes the different steps in image analysis. Figure 3 and Video 1 shows an example of a 45-y-old patient with colorectal cancer undergoing semiautomated nodal staging. In this particular patient, MRI identified six positive lymph nodes (< 10 mm each), reconstructed as a 3D dataset, whereas all positive lymph nodes were missed by PET scans. Figure 4 and Video 2 show reconstructions and analyses from a patient with a breast cancer primary with bilateral nodal metastases. Note the high spatial resolution allowing the detection of a 3-mm nodal metastasis.

**Video 2:** Automated 3D Reconstruction of Axillary Nodal Anatomy

## Discussion

We show that it is feasible to extract various quantitative tissue parameters to predict the likelihood of nodal metastases in vivo. These results are highly relevant in cancer staging because they provide evidence that (a) quantitative tissue parameters enable diagnosis of lymph node metastases while reducing interobserver variability and (b) that semiautomated reconstructions allow spatially more extensive mapping than is currently possible.

Metastases to lymph nodes occur during growth of most primary malignancies, and their presence mandates the need for more extensive and systemic therapy. Nodal cancer staging currently relies on invasive procedures (surgical lymph node dissection, sentinel lymph node resection, biopsy) with significant morbidity and cost [22,23], or insensitive tomographic imaging methods [24]. For example, detection sensitivities using size criteria with state-of-the-art multislice CT are as low as 50%, whereas PET imaging of nonenlarged nodes has equally low sensitivities [14]. Based on the observation that nanoparticulate solutions accumulate in nodal macrophages upon systemic injections [25,26], lymphotropic superparamagnetic preparations have been developed [16]. In earlier clinical trials (using lower spatial resolution sequences), metastases of 1–2 mm have been detected [17], whereas as few as 1,000 tumor cells have been detected in nodes in experimental mouse models [18]. Despite these advances, it has been difficult to acquire images of sufficiently high resolution and to derive parameters to automate diagnosis. The data presented here indicate that unique magnetic parameters allow identification of nodal metastases and accurate 3D reconstructions, including surgically inaccessible lymph nodes.

The significance of the above findings is 3-fold. First, the ability to directly and noninvasively monitor nodal tumor involvement represents a powerful diagnostic tool for cancer. Accurate staging represents the cornerstone for triaging patients to either localized or to more aggressive and systemic therapies. Second, the method described here was sensitive for the limited subsets of primary cancers tested. It is reasonable to hypothesize that such analysis could be applied to staging of other common primaries. In particular, lung, colorectal, genitourinary, and head and neck cancers could benefit from this staging procedure. In addition to nodal staging, the nanoparticle-enhanced MRI can also be used to measure microvascularity in primary tumors [27] and to improve the detection of liver metastases [28]. Third, our results are significant because the semiautomated staging method is highly accurate and reduces variability in visual image analyses between different observers.

The LMRI staging technique is believed to be clinically relevant in several key areas. First, LMRI may play a significant role in avoiding unnecessary surgeries, that is, those in node-positive patients. Second, since LMRI can detect lymph nodes outside traditional surgical fields, this information may influence surgical approaches. In colorectal cancer, LMRI may provide a “sentinel-node-like” guide to staging. Third, it is likely that LMRI would be useful to identify appropriate patients to receive neoadjuvant chemotherapy prior to surgery. Currently, neoadjuvant therapy is often reserved for postoperative patients, once the nodal status has been determined. Fourth, LMRI may be particularly useful to guide radiation therapy by mapping the complete nodal status onto bony and vascular landmarks. Finally, LMRI could be used to avoid invasive diagnostic procedures, which are not part of therapy. For example, LMRI could replace lymphangiography, mediastinoscopy, or endoscopic ultrasound for nodal staging.

Our findings have a number of direct implications for technology development and in clinical care. Accurate measurements of T2* relies on motion artifact-free multiecho pulse sequences that are not routinely available on clinical scanners at spatial resolutions required for nodal staging. Such sequences will have to be implemented and combined with postprocessing tools to simplify and semiautomate analysis. Similar software approaches are already used routinely in lung nodule characterization [29] or screening for breast cancers [30]. We predict that in the case of LMRI, such automation routines will be highly specific, given the unique mechanism of image contrast. As a proof-of-principle, we implemented approaches to identify, segment, analyze, and display nodal information. While the current technology is already highly accurate, we anticipate further improvements with hardware and software advances. We hope that this will ultimately translate into clinical practice and replace unnecessary intervention.

**Patient Summary**

**Background**

When deciding on treatment for patients with cancer, it is very important to assess whether the cancer has spread to lymph nodes—both to help decide what treatment a patient should have and what the eventual outcome might be. Previous ways of finding involved lymph nodes included taking out the nodes by surgery, ultrasound, and CT and MRI scans.

**What Does This Study Show?**

A solution of magnetic nanoparticles that tend to go to lymphoid organs was injected and then tracked by MRI. The pattern of the particles was abnormal when there was metastasis in the nodes, and it was possible to train a computer to recognize this abnormality. The authors developed the program in one group of patients and then tested it in another group, in which they were able to correctly predict whether the nodes were involved in about nine of ten nodes. In addition, they could use the information to display a virtual picture of the involved nodes.

**What Does This Study Mean for Patients?**

The technique will need to be validated in a larger group of patients, and by other investigators. However, it means that it is potentially possible to work out much more precisely, and with less chance of error, whether lymph nodes are involved in cancer. Hence, treatment can be better planned, and if surgery is needed to remove nodes for analysis, then this technique could ensure that the surgery is as minimal as possible.

**Where Can I Get More Information?**

RadiologyInfo, a public information site developed by the American College of Radiology and the Radiological Society of North America: http://www.radiologyinfo.org/

Medline Plus, which has health information from the National Library of Medicine: http://www.nlm.nih.gov/medlineplus/cancer.html

## Abbreviations

CT: computed tomography
LNM: lymph node to muscle ratio
LMRI: lymphotropic nanoparticle enhanced magnetic resonance imaging
MIP: maximum intensity projection
MRI: magnetic resonance imaging
PET: positron-emission tomography
ROI: region of interest
SI: signal intensity
SNR: signal-to-noise ratio
3D: three-dimensional
